# Pituitary stalk interruption syndrome presenting as short stature: a case report

**DOI:** 10.1186/1752-1947-8-445

**Published:** 2014-12-19

**Authors:** Nanik Ram, Syed Ahsan Ali, Syed Zubair Hussain

**Affiliations:** 54 Section of Endocrinology, Department of Medicine, The Aga Khan University Hospital, Stadium Road, Karachi, 74800 Pakistan; 55 Section of Internal Medicine, Department of Medicine, The Aga Khan University Hospital, Stadium Road, Karachi, 74800 Pakistan

**Keywords:** PSIS, Short stature, Pan hypopituitarism

## Abstract

**Introduction:**

Pituitary stalk interruption syndrome is a rare congenital abnormality of the pituitary that is responsible for anterior pituitary deficiency. It is characterized by a classic triad of interrupted pituitary stalk, absent or ectopic posterior pituitary, and anterior pituitary hypoplasia or aplasia. Clinical presentation varies according to age. In adults it presents as short stature and anterior pituitary deficiency. Without early diagnosis and treatment, mortality and morbidity in these patients is high. Early diagnosis and treatment of this rare disease can prevent permanent short statue of the patient. We report the first case of pituitary stalk interruption syndrome from Pakistan.

**Case presentation:**

A 17-year-old Pakistani young man presented with short stature and underdeveloped secondary sexual characters. His siblings and parents were healthy, with normal height. An examination showed his blood pressure was 90/60mmHg, and his height, weight, and body mass index were 142cm, 34.5kg, and 17.10kg/m2, respectively. He had no hair growth on his face, axilla, or pubis. His testes were between 1 and 2mL in size, with a 4cm-at-stretch micropenis. His lab investigations showed that his thyroid stimulating hormone (TSH) was 8.58uIU/mL (0.4 to 4.2), his free thyroid hormone level FT4 was 0.46ng/dL (0.89 to 1.76), his prolactin was 21.1ng/mL (3.0 to 14.7), and his baseline cortisol was 0.30ug/dL (4.3 to 22.4). His cortisol level after 60 minutes of cosyntropin injection was 3.5ug/dL (4.3 to 22.4), his insulin like growth factor IGF-1 was 31.56ng/mL (247.3 to 481.7), his testosterone level was under 2.5ng/dL (2 to 800), his follicle stimulating hormone FSH was 0.41uIU/mL (0.0 to 10.0), and his leutinizing hormone LH was under 0.1uIU/mL (1.2 to 7.8). His bone age was 10 years according to the Greulich and Pyle method, as shown by X-rays. The results from his pituitary magnetic resonance imaging scan were consistent with pituitary stalk interruption syndrome.

**Conclusions:**

We describe a young man who presented with short stature and was found to have pituitary stalk interruption syndrome. Despite the fact that this is a rare disorder, it should always be kept in the differential diagnosis of a patient presenting with short stature. Patients with this disease have an excellent opportunity to reach normal height if they present before the joining of epiphyses.

## Introduction

Pituitary stalk interruption syndrome (PSIS) is a rare congenital abnormality of the pituitary that is responsible for anterior pituitary deficiency. It was first reported by Fujisawa *et al.* [1]. It is associated with either isolated growth hormone deficiency (GHD) or multiple anterior pituitary hormone deficiencies (MPHD) - growth hormone deficiency associated with abnormality of at least one of the other anterior pituitary hormones, with normal function of posterior pituitary [2]. Although the exact prevalence of PSIS is uncertain, a recently published report showed the estimated incidence of 0.5 in every 1,000,000 births [3].

PSIS is characterized by a classic triad of interrupted pituitary stalk, absent or ectopic posterior pituitary, and anterior pituitary hypoplasia or aplasia [4]. Clinical presentation of PSIS depends on the age at the time of diagnosis. In neonates it presents as neonatal hypoglycemia, prolonged neonatal (physiological) jaundice, cryptorchidism, and micropenis. In older children and adults it is characterized by short stature [2, 5]. Diagnosis of PSIS can be suspected from clinical findings, but a magnetic resonance imaging (MRI) scan can provide a definitive diagnosis. MRI findings include hypoplasia or aplasia of the anterior pituitary, absence of the hyperintense posterior lobe within the sella turcica and its presence at the level of the median eminence or at the pituitary stalk level as a hyperintense nodule, and absent or thinned out pituitary stalk [3, 4, 6, 7].

Importance of early diagnosis of pituitary hormone deficiencies is twofold. Firstly, if it remains untreated it is associated with substantial mortality and morbidity. Secondly, insufficient height at the onset of puberty leads to short final height. Early diagnosis and treatment of GHD is necessary to allow growth to reach a normal height before puberty [8–10]. Most data on this syndrome is from Western countries. Apart from China, no significant data is available from Asian countries [3]. Here, we present the case of a young man who presented to us with short stature and ultimately was found to have PSIS. To best of our knowledge, this is the first ever reported case of PSIS from Pakistan.

## Case presentation

We report the case of a 17-year-old Pakistani young man who presented with short stature and underdeveloped secondary sexual characters. He had been operated on for patent foramen ovale five years previously and the procedure was uneventful. His birth history was found to be significant due to breech presentation. He has experienced no delay in achieving developmental milestones, his intelligence level was within the normal range, and his school records reflected good performance. He had six siblings, all of which were healthy, with normal height. His mother and father had a height of 165cm and 154cm, respectively. An examination showed that his pulse was 109/min, his blood pressure was 90/60mmHg, his height was 142cm, his weight was 34.5kg, his body mass index was 17.10kg/m^2^, and his calculated mid-parental height (MPH) was 166.5cm, with an MPH range of 156.5 to 176.5cm. His Standard Deviation Score (SDS) was - 4.3. He had no hair growth on his face, axilla, and pubis (Tanner stage 1). Both of his testes were between 1 and 2mL in volume, and his stretch penile length was 4cm. A systemic examination was unremarkable.

His lab investigations showed a fasting blood glucose level of 85mg/dL, sodium at 141mmol/L, potassium at 3.8mmol/L, serum calcium at 9.4mg/dL, phosphorus at 6.2mg/dL (normal, 2.5 to 4.5mg/dL), albumin at 4.5gm/dL, hemoglobin at 11.4g/dL, thyroid stimulating hormone (TSH) at 8.58uIU/mL, free thyroxine at (FT4) 0.46ng/dL (normal, 0.89 to 1.76ng/dL), an anti-thyroid peroxidase antibody (anti-TPO) level of 17.8IU/mL (normal, less than 35IU/mL), and an anti-thyroglobulin antibodies level of under 20IU/mL (normal, less than 40IU/mL), prolactin at 21.1ng/mL (normal, 3.0 to 14.7ng/mL). A short synacthen test revealed a morning baseline cortisol level of 0.30ug/dL (normal, 4.3 to 22.4ug/dL), and cortisol after 60 minutes of 250mcg cosyntropin injection was 3.5ug/dL. His insulin like growth factor-1 (IGF-1) level was 31.56ng/mL (normal, 247.3 to 481.7ng/mL), his morning testosterone level was under 2.5ng/dL (normal, 280 to 800ng/dL), his follicle stimulating hormone level (FSH) was 0.41uIU/mL (normal, 0.0 to 10.0), and his luteinizing hormone level (LH) was under 0.1uIU/mL (normal 1.2 to 7.8).

A biochemical picture favored an anterior pituitary hormones deficiency. Symptoms of diabetes insipidus such as polyuria, polydipsia, nocturia, and nocturnal enuresis were absent. His 24-hour urinary volume was 1800ml and his serum osmolarity was 285mosm/kg.

**Figure 1:**
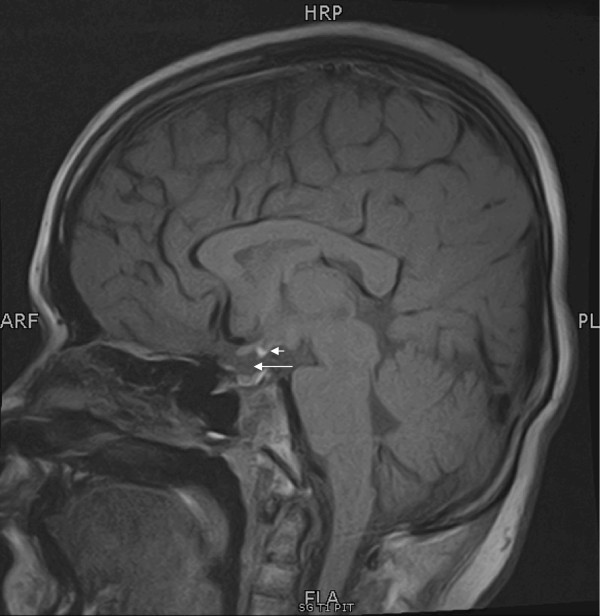
**T1 weighted magnetic resonance image of the pituitary.** Pituitary stalk is absent (long arrow). Posterior pituitary is not seen in relation to the anterior pituitary in the pituitary fossa and is seen as hyperintense focus at the median eminence (short arrow). Anterior pituitary is seen in pituitary fossa at its normal location and returning a normal signal.

His bone age was 10 years according to the Greulich and Pyle method as was shown by his X-rays. A magnetic resonance imaging (MRI) scan of his pituitary showed T1 hyperintense focus in the midline at the median eminence with non-visualization of pituitary stalk. Posterior pituitary was not seen in relation to the anterior pituitary in the pituitary fossa. Anterior pituitary was seen in pituitary fossa at its normal location and was returning a normal signal. This represented ectopic posterior pituitary with absent pituitary stalk (Figure 1).

## Discussion

PSIS is a rare congenital syndrome characterized by a classic triad of interrupted pituitary stalk, absent or ectopic posterior pituitary, and anterior pituitary hypoplasia or aplasia. The pathogenesis of PSIS is still unknown. A significant proportion of patients with congenital deficiency of pituitary hormones and PSIS are more likely to have been delivered by breech or Cesarean section and have suffered from neonatal hypoxemia than the general population. Breech delivery leads to obvious deformation of head which can result in injury to the pituitary stalk. Similarly, hypoxemia due to anoxia after birth may also lead to injury of the pituitary stalk and pituitary. This has led to the belief by some investigators that PSIS is secondary to these events leading to injury to this region during birth. However, the association of this syndrome with micropenis and cryptorchidism, as well as the occurrence of familial or syndromal forms, suggests an antenatal origin of its pathogenesis. Thus the abnormal birth data are a consequence and not a cause of PSIS. Abnormal *HESX1*, *LHX4*, *SOX3*, and *PROKR2* genes have been reported in rare cases of PSIS [3, 4, 9, 11]. Between 20 and 50% of patients with PSIS also have associated congenital malformations. These malformations include mainly the structures or organs along the midline (for example, cleft lip, absence of diaphragm, hypoplasia of optic nerve, bulging brain, or harelip) which indicates that the gene defect corresponding to this disease may be related to the genes responsible for embryonic development of the hypothalamus-pituitary area. Moreover, those having isolated GHD have a higher risk of congenital malformations as compared to those having multiple anterior pituitary deficiencies [3, 4]. Our patient also had patent foramen ovale. To the best of our knowledge, this association of PSIS has never been reported in literature.

Due to the limited number of reported cases of PSIS, clinical manifestations of this disease are complex and diverse. Prolonged neonatal jaundice, hypoglycemia cryptorchidism, and micropenis are common presentations in neonates. On the other hand, growth retardation is the most common presentation in older children and adults. Most patients lack sexual development [2, 4, 5]. Our patient presented at adult age with short stature and lack of sexual development. PSIS is a male predominant disease [3, 5]. Age at the time of diagnosis has great variation in the literature. The age of our patient at diagnosis was 17 years, which is much older than mean age of 3.6 years reported by Gascoin-Lachambre *et al.* [10], 4.0 years by Pinto *et al*. [4], and the 9.6 years reported by Reynaud *et al*. [5]. However, Guo *et al*. [3] reported a median age of 19.7 years in his series which is closer to the age of our patient. The age for diagnosis of symptomatic PSIS in various studies suggests an important delay in the diagnosis. Interestingly, PSIS may progress from isolated GHD to multiple pituitary hormone deficiencies even during the second or third decade of life. Similarly, our patient underwent a major surgical intervention without developing adrenal crisis. Close follow-up of such patients for monitoring of other hormonal deficiencies is essential if they initially present with isolated GHD [2].

Our patient’s thyroid function tests favored secondary hypothyroidism because of a very low FT4 level and slightly increased TSH level, together with a negative anti-thyroid antibodies level; Guo *et al.* [3] also reported such thyroid function test results in a subgroup of PSIS patients [3]. He is currently on hydrocortisone, thyroxine, growth hormone, and low-dose sex steroids replacement for somatic and sexual growth.

MRI findings of PSIS include hypoplasia or aplasia of anterior pituitary, absence of the hyper intense posterior lobe within the sella turcica and its presence at the level of the median eminence or at the pituitary stalk level as a hyper intense nodule, and absent or thinned out pituitary stalk [3, 4, 6, 7]. However, there are variations in MRI appearances of this syndrome. These variations include the height of the anterior pituitary (from absence to normal), the appearance of the posterior pituitary lobe (ectopic at the base of the hypothalamus or along the pituitary stalk, absent, or normal), and the form of the pituitary stalk (interrupted, thin, absent, or normal). The abnormality can be limited to an ectopic posterior pituitary [4].

## Conclusions

We report the case of a young man who presented with short stature and ultimately was found to have a rare congenital syndrome called PSIS. Despite the fact that PSIS is a rare disorder it should always be kept in the differential diagnosis of a patient presenting with short stature. Patients with this disease have an excellent opportunity to reach their normal height if they present before the joining of epiphyses.

## Consent

Written informed consent was obtained from the patient’s legal guardian(s) for publication of this case report and any accompanying images. A copy of the written consent is available for review by the Editor-in-Chief of this journal.

## Abbreviations

FSH: Follicle stimulating hormone level
FT4: Free thyroxine
GHD: Growth hormone deficiency
IGF-1: Insulin like growth factor-1
LH: Luteinizing hormone level
MRI: Magnetic resonance imaging
PSIS: Pituitary stalk interruption syndrome
TSH: Thyroid stimulating hormone.
